# Supplementary material for: Underpinning heterogeneity in synaptic transmission by presynaptic ensembles of distinct morphological modules
Source: Nat Commun. 2019 Feb 18;10:826. doi: 10.1038/s41467-019-08452-2 (PMC6379440; doi:10.1038/s41467-019-08452-2)
Supplement: Supplementary file 1 — Supplementary Information [file 41467_2019_8452_MOESM1_ESM.pdf]

SUPPLEMENTARY INFORMATION

Underpinning heterogeneity in synaptic transmission by presynaptic ensembles of distinct morphological modules

Fekete et al.

SUPPLEMENTARY FIGURES AND LEGENDS

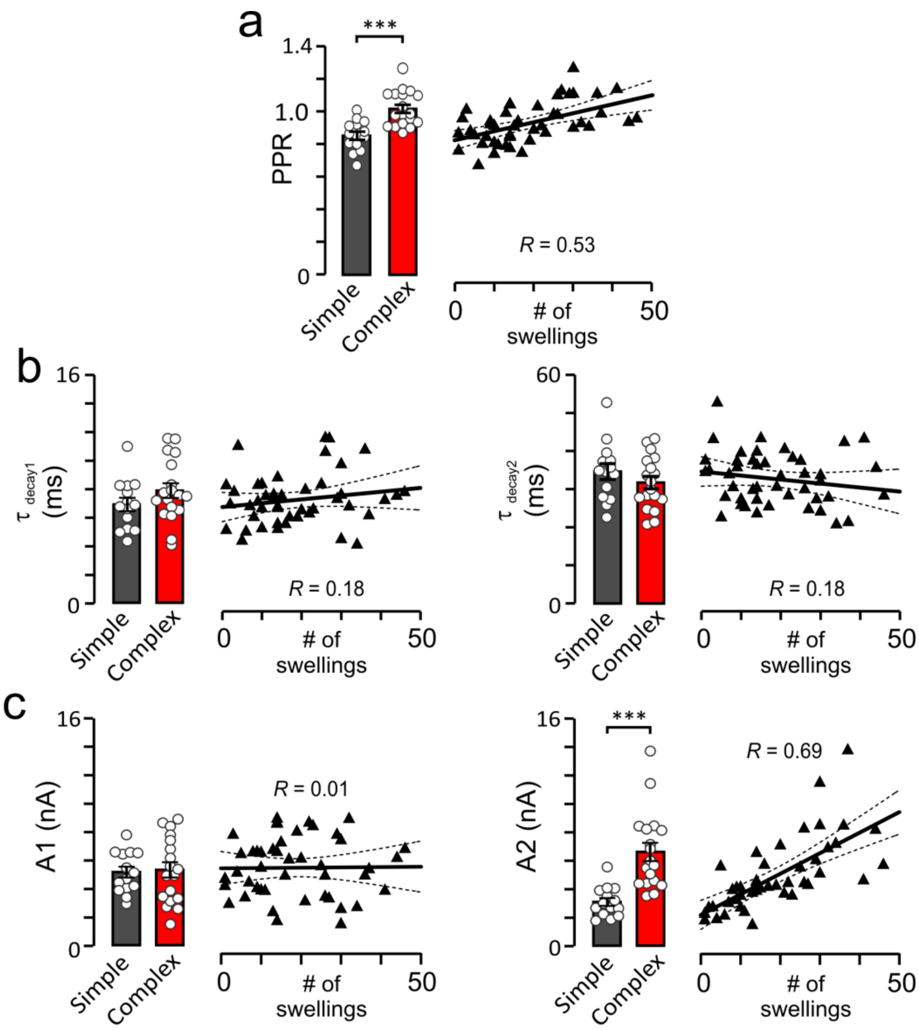

Supplementary Figure 1. Short-term synaptic plasticity correlates with the complexity of calyx morphology. a-c, Bar graphs (left) show the parameters of short-term synaptic plasticity (STP) in simple and complex calyces. Scatter-plots of all recorded synapses with linear regression represent correlation between STP and calyx complexity (right, black triangles; mean ± 95 % confidence interval). **a,** Paired-pulse ratio (PPR, simple, 0.853 ± 0.025, n = 14/11, complex, 1.017 ± 0.026, n = 18/16, t = 4.518, $p < 0.0001$, df = 30, unpaired t-test [u.t.t.]). **b,** τ_{decay1} and τ_{decay2} are decay time constants, **c,** A1 and A2 are amplitudes of the fast- and slowly releasing subpools of SVs, respectively. The decay of EPSC amplitudes (300 Hz, 200 ms) was fitted by double exponential function (τ_{decay1}, simple, 6.90 ± 0.47 ms, n = 14/11, complex, 7.88 ± 0.52 ms, n = 18/16, t = 1.361, p = 0.184, df = 30, u.t.t.; τ_{decay2}, simple, 34.52 ± 2.07 ms, n = 14/11, complex, 31.64 ± 1.64 ms, n = 18/16, t = 1.106, p = 0.278, df = 30, u.t.t.; A1, simple, 5.16 ± 0.39 nA, n = 14/11, complex, 5.32 ± 0.54 nA, n = 18/16, t = 0.2327, p = 0.818, df = 28, unpaired t-test with Welch correction [u.t.t.W.]; A2, simple, 3.09 ± 0.28 nA, n = 14/11, complex, 6.62 ± 0.65 nA, n = 18/16, t = 5.014, $p < 0.0001$, df = 22, u.t.t.W.). R demonstrates correlation strength. Bar graphs summarize mean ± SEM (***, $p < 0.001$).

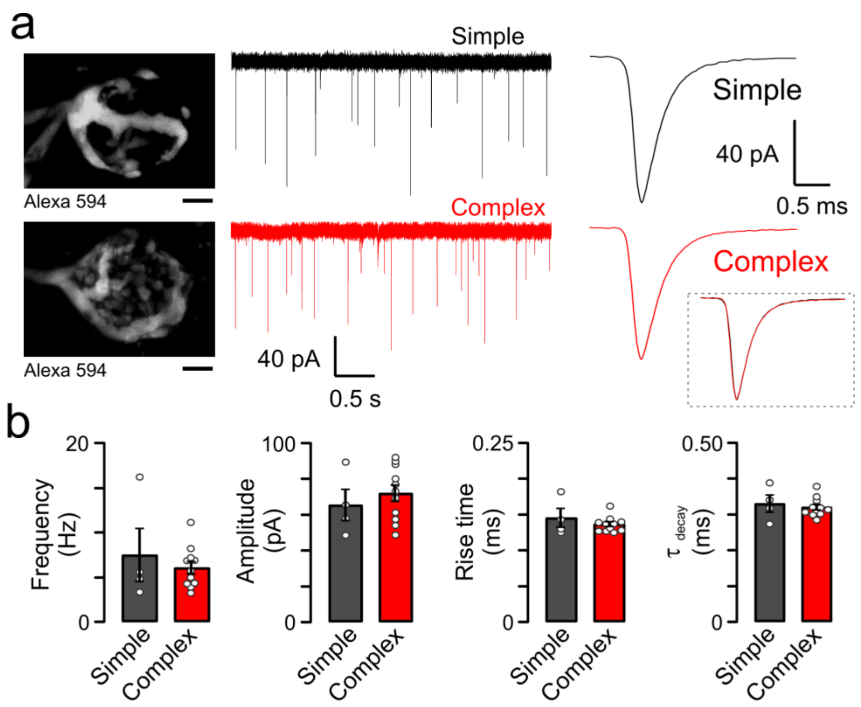

Supplementary Figure 2. Gating of postsynaptic glutamate receptors is similar in simple and complex calyces. a, Representative miniature EPSC (mEPSC) recordings from simple (black trace) and complex (red trace) calyces are shown (left, z-axis projection images; middle, example traces; right, average events). Inset depicts the amplitude-scaled, superimposed average mEPSCs. **b,** Bar graphs (mean ± SEM) summarize frequency, amplitude, rise time and decay time (τ_{decay}) of mEPSCs in simple and complex calyces (frequency, simple, 7.4 ± 2.9 Hz, n = 4/3, complex, 6.0 ± 0.7 Hz, n = 11/8, t = 0.466, p = 0.673, df = 3, unpaired t-test with Welch correction [u.t.t.W.]; amplitude, simple, 65.2 ± 8.7 pA, n = 4/3, complex, 71.8 ± 4.6 pA, n = 11/8, t = 0.716, p = 0.487, df = 13, unpaired t-test [u.t.t.]; rise time, simple, 0.145 ± 0.013 ms, n = 4/3, complex, 0.136 ± 0.003 ms, n = 11/8, t = 0.670, p = 0.551, df = 3, u.t.t.W.; τ_{decay}, simple, 0.33 ± 0.02 ms, n = 4/3, complex, 0.32 ± 0.01 ms, n = 11/8, t = 0.521, p = 0.611, df = 13, u.t.t.). Scale bars: 5 µm (**a**).

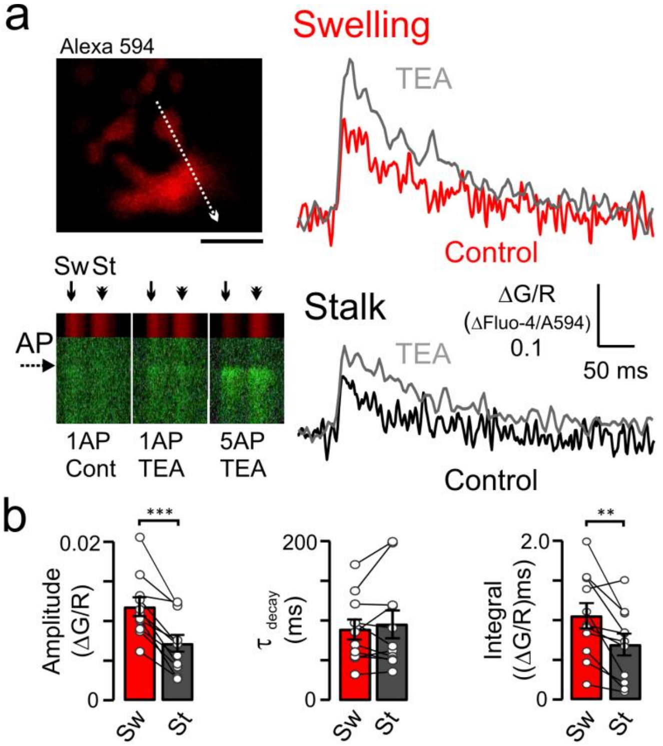

Supplementary Figure 3. Single action potential-evoked Ca^{2+} transients are heterogeneous throughout the calyx. a, Representative calyx was loaded with Fluo-4 (50 μM) and Alexa 594 (15 μM) then line-scanned (see arrow across a swelling and neighboring stalk). Line-scans show action potential-evoked Ca^{2+} transients. As positive control, we applied TEA (1 mM; high-threshold K^+-channel blocker) to broaden APs, hence to increase Ca^{2+} influx. Single AP-evoked Ca^{2+} transients were larger in swellings than stalks (red trace, control swelling; black trace, control stalk; grey traces, TEA). **b,** Bar graphs (mean ± SEM; **, $p < 0.01$, ***, $p < 0.001$) summarize the amplitude, decay time constant (τ_{decay}) and integral (amplitude × τ_{decay}) of Ca^{2+} transients in swellings (Sw, red) and stalks (St, black; amplitude [ΔG/R], swelling, 0.0117 ± 0.0012, n = 11/9, stalk, 0.0071 ± 0.0011, n = 11/9, t = 6.619, p < 0.0001, df = 10, paired t-test [p.t.t.]; τ_{decay}, swelling, 88.63 ± 12.42 ms, n = 11/9, stalk, 94.85 ± 17.44 ms, n = 11/9, t = 0.812, p = 0.436, df = 10, p.t.t.; integral [(ΔG/R)ms], swelling, 1.049 ± 0.159, n = 11/9, stalk, 0.688 ± 0.137, n = 11/9, t = 3.678, p = 0.0043, df = 10, p.t.t.). Scale bar: 5 μm (**a**).

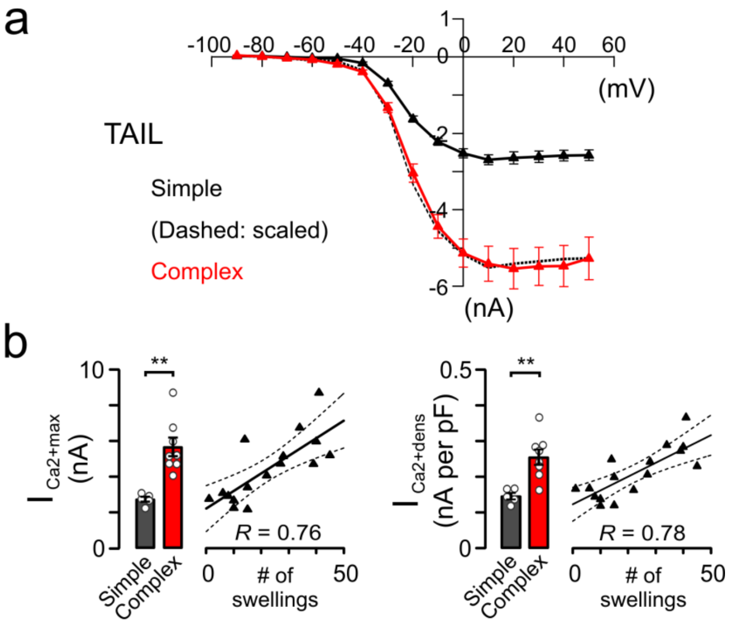

Supplementary Figure 4. Tail Ca^{2+} current density is larger in complex calyces. a, Presynaptic Ca^{2+} currents (I_{Ca2+}) were recorded from simple (black trace) and complex (red trace) calyces kept at -100 mV. I_{Ca2+} were tested by voltage steps between -90 and +50 mV with 10 mV increments. Average voltage-dependence curves of tail I_{Ca2+} recorded from simple and complex calyces (mean ± SEM) are shown. Amplitude-scaled version of simple calyx I-V curve (dashed black trace) overlaps with the complex one. **b**, Maximal tail I_{Ca2+} amplitude ($I_{Ca2+max}$) and tail I_{Ca2+} density ($I_{Ca2+dens}$) of simple and complex calyces are shown by the bar graphs (tail I_{Ca2+} amplitude, simple, 2.73 ± 0.14 nA, n = 5/4, complex, 5.65 ± 0.52 nA, n = 8/7, t = 5.398, p =0.001, df = 7, unpaired t-test with Welch correction [u.t.t.W.]; tail I_{Ca2+} density, simple, 0.146 ± 0.009 nA per pF, n = 5/4, complex, 0.255 ± 0.022 nA per pF, n = 8/7, t = 4.665, p =0.001, df = 9, u.t.t.W.). Dot-plots of all recorded calyces (right; black triangles) were fitted with linear function (mean ± 95 % confidence interval). R demonstrates correlation strength. Bar graphs summarize mean ± SEM (**, p < 0.01).

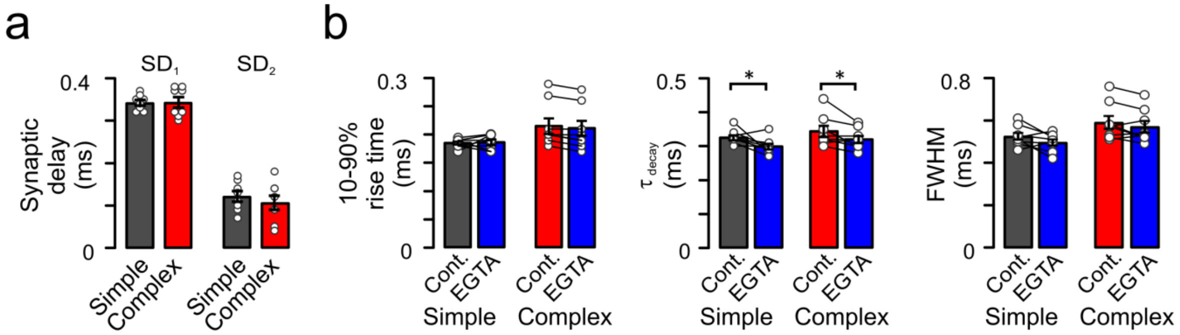

Supplementary Figure 5. Synaptic delays and EPSC kinetics are similar in simple and complex calyces. Bar graphs (mean ± SEM; *, $p < 0.05$) summarize, **a,** synaptic delays (SD_1 and SD_2), **b,** EPSC time course (10-90% rise time; τ_{decay}, decay time constant; FWHM, full width at half maximum) in simple (black, control; n = 8/7) and complex calyces (red, control; n = 8/8) and those after EGTA injection (blue; simple, n = 8/7; complex, n = 8/8). The difference in EGTA effect on simple and complex calyces was quantified as EGTA/Control (see Supplementary Table 1).

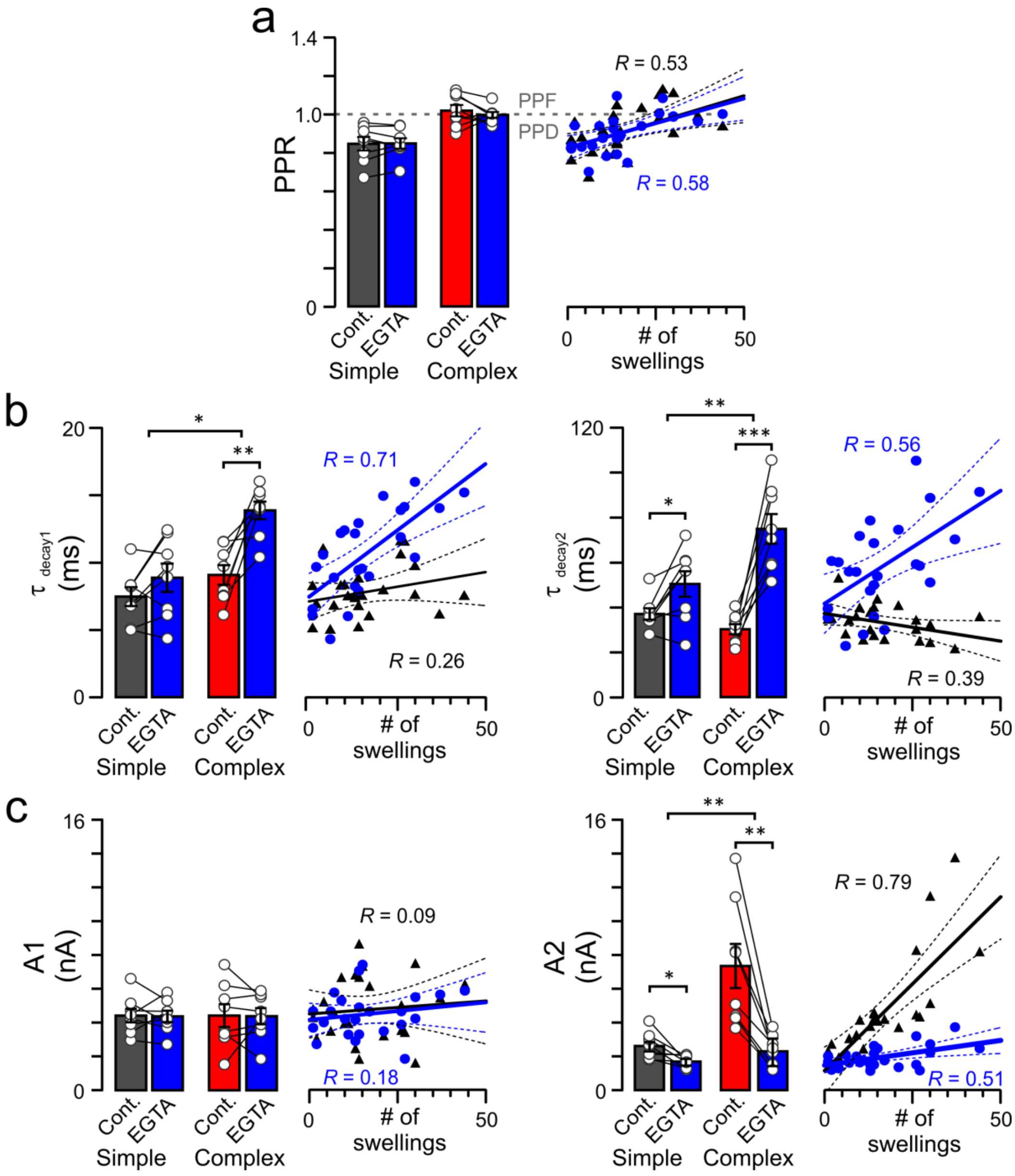

Supplementary Figure 6. EGTA sensitivity of short-term synaptic plasticity correlates with the complexity of calyx morphology. a-c, Bar graphs (left) show short-term synaptic plasticity (STP) in simple (black, control) and complex (red, control) calyces, and after presynaptic EGTA loading (blue; 10 mM, 3-5 min). Scatter-plots of all recorded synapses with linear regression represent correlation between STP and calyx complexity (right; black triangles, control; blue dots, EGTA; mean ± 95 % confidence interval). **a,** Paired-pulse ratio (PPR, simple, control, 0.847 ± 0.035, EGTA, 0.848 ± 0.027, n = 8/7, t = 0.123, p = 0.906, df = 7, paired t-test [p.t.t.]; complex, control, 1.019 ± 0.030, EGTA, 0.997 ± 0.015, n = 8/8, t = 0.814, p = 0.442, df = 7, p.t.t.). The difference in EGTA effect on simple and complex calyces was quantified as EGTA/Control (simple, 1.006 ± 0.017, n = 8/7, complex, 0.983 ± 0.027, n = 8/8, t = 0.703, p = 0.494, df = 14, unpaired t-test [u.t.t.]). **b,** τ_{decay1} and τ_{decay2} are decay time constants, **c,** A1 and A2 are amplitudes of the fast- and slowly releasing subpools of SVs, respectively. The decay of EPSC amplitudes (300 Hz, 200 ms) was fitted by double exponential function (see Supplementary Table 2). The difference in EGTA effect on simple and complex calyces was quantified as EGTA/Control (τ_{decay1}, simple,

1.190 ± 0.099, n = 8/7, complex, 1.602 ± 0.155, n = 8/8, t = 2.232, p = 0.043, df = 14, u.t.t.; τ_{decay2}, simple, 1.357 ± 0.136, n = 8/7, complex, 2.584 ± 0.295, n = 8/8, t = 3.787, p = 0.004, df = 9, unpaired t-test with Welch correction [u.t.t.W.]; A1, simple, 1.013 ± 0.077, n = 8/7, complex, 1.155 ± 0.241, n = 8/8, t = 0.548, p = 0.599, df = 8, u.t.t.W.; A2, simple, 0.694 ± 0.081, n = 8/7, complex, 0.336 ± 0.036, n = 8/8, t = 4.012, p = 0.003, df = 9, u.t.t.W.). R demonstrates correlation strength. Bar graphs summarize mean ± SEM (*, p < 0.05, **, p < 0.01, ***, p < 0.001).

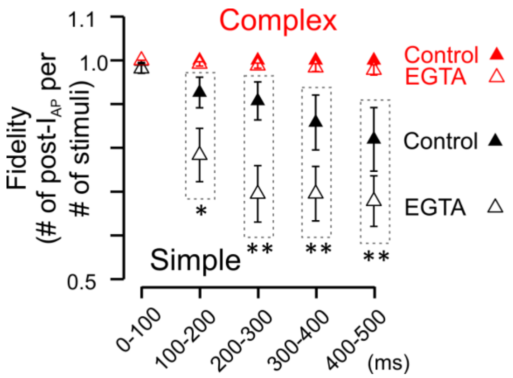

Supplementary Figure 7. Simple calyces fail early during a long-lasting train of action potentials. The time course of fidelity (calculated as the number of successful postsynaptic compound action potential currents [post-I_{AP}] per number of stimuli) in mature calyces with different morphologies is shown under control conditions and after presynaptic EGTA administration (10 mM, 3-5 min; black filled triangle, simple control; red filled triangle, complex control; black empty triangle, simple EGTA; red empty triangle, complex EGTA). Dashed rectangles and asterisks represent significant effect of EGTA on fidelity during a 100 ms segment of the 500 ms long 300 Hz stimulus train (e.g. fidelity between 100 and 200 ms, simple, control, 0.927 ± 0.035, EGTA, 0.784 ± 0.061, n = 5/5, t = 5.03, p = 0.007, df = 4, paired t-test [p.t.t.]; complex, control, 1.00 ± 0.00, EGTA, 0.992 ± 0.005, n = 8/8, t = 1.614, p = 0.151, df = 7, p.t.t.). Graphs summarize mean ± SEM (*, $p < 0.05$, **, $p < 0.01$).

SUPPLEMENTARY TABLES AND LEGENDS

Parameters	Simple (Mean ± SEM)	n	Complex (Mean ± SEM)	n	Statistical Analyses	df	t-value	P-value
Synaptic $delay_1$, SD_1 (ms)	0.343 ± 0.006	8/7	0.344 ± 0.012	8/8	unpaired t-test	14	0.091	0.929, ns
Synaptic $delay_2$, SD_2 (ms)	0.121 ± 0.012	8/7	0.106 ± 0.017	8/8	unpaired t-test	14	0.718	0.485, ns
10-90 % rise time (ms), Control	0.186 ± 0.003	8/7	0.216 ± 0.014	8/8	unpaired t-test with Welch correction	7	2.109	0.073, ns
τ_{decay} (ms), Control	0.327 ± 0.008	8/7	0.345 ± 0.016	8/8	unpaired t-test with Welch correction	9	1.009	0.340, ns
FWHM (ms), Control	0.525 ± 0.017	8/7	0.591 ± 0.031	8/8	unpaired t-test	14	1.871	0.082, ns
10-90 % rise time (ms), EGTA	0.187 ± 0.005	8/7			paired t-test (vs. control)	7	0.120	0.908, ns
10-90 % rise time (ms), EGTA			0.212 ± 0.013	8/8	paired t-test (vs. control)	7	1.691	0.135, ns
τ_{decay} (ms), EGTA	0.300 ± 0.008	8/7			paired t-test (vs. control)	7	2.432	0.045, *
τ_{decay} (ms), EGTA			0.321 ± 0.011	8/8	paired t-test (vs. control)	7	2.596	0.036, *
FWHM (ms), EGTA	0.496 ± 0.015	8/7			paired t-test (vs. control)	7	1.695	0.134, ns
FWHM (ms), EGTA			0.571 ± 0.027	8/8	paired t-test (vs. control)	7	1.581	0.158, ns
EGTA/Control, 10-90 % rise time	1.006 ± 0.029	8/7			unpaired t-test with Welch correction	8	0.812	0.440, ns
			0.982 ± 0.010	8/8				
EGTA/Control, τ_{decay}	0.921 ± 0.031	8/7			unpaired t-test	14	0.386	0.705, ns
			0.936 ± 0.023	8/8				
EGTA/Control, FWHM	0.949 ± 0.032	8/7			unpaired t-test	14	0.524	0.609, ns
			0.969 ± 0.022	8/8				

Supplementary Table 1. Summary of the synaptic delays and EPSC kinetics in mature calyces. Synaptic delays were defined as interval between $peak_1$ of pre-I_{AP} (presynaptic compound action potential currents) and EPSC onset (SD_1) or $peak_2$ of pre-I_{AP} and EPSC onset (SD_2). EGTA effects are expressed as EGTA/Control. The n shows the number of experiments (cells/animals). Data are expressed as mean ± SEM. Degrees of freedom (df), t- and p-values, and significance levels (ns, not significant, *, $p < 0.05$) are detailed for each statistics. (FWHM, full width at half maximum). See Supplementary Figure 5.

Parameters	Simple (Mean ± SEM), n	Paired t-test (df / t-value / p-value)	Complex (Mean ± SEM), n	Paired t-test (df / t-value / p-value)
τ_{decay1} (ms), control	7.44 ± 0.69, 8/7	7/1.833/0.1095, ns	9.06 ± 0.73, 8/8	7/4.769/0.002, **
τ_{decay1} (ms), EGTA	8.85 ± 1.04, 8/7		13.83 ± 0.65, 8/8	
τ_{decay2} (ms), control	37.09 ± 2.53, 8/7	7/2.763/0.028, *	30.26 ± 2.31, 8/8	7/6.405/0.0004, ***
τ_{decay2} (ms), EGTA	50.46 ± 5.69, 8/7		75.09 ± 6.67, 8/8	
A1 (nA), control	4.42 ± 0.40, 8/7	7/0.133/0.8981, ns	4.42 ± 0.67, 8/8	7/0.068/0.9479, ns
A1 (nA), EGTA	4.38 ± 0.34, 8/7		4.39 ± 0.48, 8/8	
A2 (nA), control	2.61 ± 0.28, 8/7	7/3.090/0.0176, *	7.35 ± 1.31, 8/8	7/4.678/0.0023, **
A2 (nA), EGTA	1.69 ± 0.12, 8/7		2.29 ± 0.32, 8/8	

Supplementary Table 2. EGTA effect on the decay times and amplitudes of synaptic vesicle subpools. We fitted the decay of EPSC amplitudes (300 Hz, 200 ms) before and after presynaptic EGTA application (10 mM, 3-5 min) with a double exponential function. τ_{decay1}, τ_{decay2}, A1 and A2 represent the fast and slow decay time constants and amplitude components of SV depletion, respectively, in simple and complex calyces. The n shows the number of experiments (cells/animals). Data are expressed as mean ± SEM. Degrees of freedom (df), t- and p-values, and significance levels (ns, not significant, *, $p < 0.05$, **, $p < 0.01$, ***, $p < 0.001$) were calculated by paired t-test. See Supplementary Figure 6.

Parameters	Stimulus Frequency (Hz)	Swelling (Mean ± SEM)	Stalk (Mean ± SEM)	Statistical Analyses	n	$F_{(1,24)}$	P-value, ANOVA	P-value, Post hoc
Amplitude (ΔG/R)	300 Hz	0.0457 ± 0.0058	0.0320 ± 0.0047	2-way repeated measures ANOVA, Bonferroni post hoc test (3 comparisons)	9/8	196.6	<0.0001, ***	<0.0001, ***
	200 Hz	0.0318 ± 0.0045	0.0212 ± 0.0035					<0.0001, ***
	100 Hz	0.0152 ± 0.0022	0.0098 ± 0.0019					0.0002, ***
Integral ((ΔG/R)ms)	300 Hz	10.42 ± 1.52	7.75 ± 1.39	2-way repeated measures ANOVA, Bonferroni post hoc test (3 comparisons)	9/8	171.9	<0.0001, ***	<0.0001, ***
	200 Hz	6.61 ± 1.12	4.86 ± 0.86					<0.0001, ***
	100 Hz	3.27 ± 0.61	2.15 ± 0.46					0.0001, ***
10-90 % rise time (ms)	300 Hz	121.7 ± 4.51	126.1 ± 5.63	2-way repeated measures ANOVA, Bonferroni post hoc test (3 comparisons)	9/8	6.399	0.0184, *	0.6236, ns
	200 Hz	119.5 ± 5.40	134.1 ± 5.06					0.1141, ns
	100 Hz	122.8 ± 8.67	142.7 ± 7.35					0.0343, ns
τ_{decay} (ms)	300 Hz	84.0 ± 5.67	97.2 ± 10.89	2-way repeated measures ANOVA, Bonferroni post hoc test (3 comparisons)	9/8	16.45	0.0005, ***	0.1198, ns
	200 Hz	68.5 ± 7.23	92.4 ± 9.25					0.0076, *
	100 H	69.4 ± 6.74	89.8 ± 6.13					0.0198, ns

Supplementary Table 3. Summary of the magnitude and kinetics of action potential train-evoked Ca^{2+} transients in swellings and stalks. Action potential (AP) trains (100-300 Hz, 200 ms) were elicited by current injection in current-clamp mode or afferent stimulation using bipolar electrode after pipette removal. The n shows the number of experiments (cells/animals). Data are expressed as mean ± SEM (ns, not significant, *, p < 0.05, ***, p < 0.001). The parameters of F distribution (dfn and dfd, degrees of freedom numerator and denominator, respectively) were used to calculate probability values for ANOVA and are given as F value indices ($F_{(dfn,dfd)}$). The p-values and significance levels (ns, not significant, *, p < 0.05, ***, p < 0.001) are detailed for each statistics. See Fig. 8.
